# Spectrum Evaluation in CR-Based Smart Healthcare Systems Using Optimizable Tree Machine Learning Approach

**DOI:** 10.3390/s23177456

**Published:** 2023-08-27

**Authors:** Ahmad Raza, Mohsin Ali, Muhammad Khurram Ehsan, Ali Hassan Sodhro

**Affiliations:** 1 Department of Computer Engineering, Khwaja Fareed University of Engineering & Information Technology, Rahim Yar Khan 64200, Pakistan; cpen18151003@kfueit.edu.pk (A.R.); mohsin.ali@kfueit.edu.pk (M.A.); 2 Faculty of Engineering Sciences, Bahria University, Islamabad 44000, Pakistan; mkehsan.buic@bahria.edu.pk; 3 Department of Computer Science, Kristianstad University, SE-29188 Kristianstad, Sweden

**Keywords:** smart healthcare, spectrum sensing, optimizable tree, machine learning, cognitive radio

## Abstract

The rapid technological advancements in the current modern world bring the attention of researchers to fast and real-time healthcare and monitoring systems. Smart healthcare is one of the best choices for this purpose, in which different on-body and off-body sensors and devices monitor and share patient data with healthcare personnel and hospitals for quick and real-time decisions about patients’ health. Cognitive radio (CR) can be very useful for effective and smart healthcare systems to send and receive patient’s health data by exploiting the primary user’s (PU) spectrum. In this paper, tree-based algorithms (TBAs) of machine learning (ML) are investigated to evaluate spectrum sensing in CR-based smart healthcare systems. The required data sets for TBAs are created based on the probability of detection ($P_{d}$) and probability of false alarm ($P_{f}$). These data sets are used to train and test the system by using fine tree, coarse tree, ensemble boosted tree, medium tree, ensemble bagged tree, ensemble RUSBoosted tree, and optimizable tree. Training and testing accuracies of all TBAs are calculated for both simulated and theoretical data sets. The comparison of training and testing accuracies of all classifiers is presented for the different numbers of received signal samples. Results depict that optimizable tree gives the best accuracy results to evaluate the spectrum sensing with minimum classification error (MCE).

## 1. Introduction

**Figure 1:**
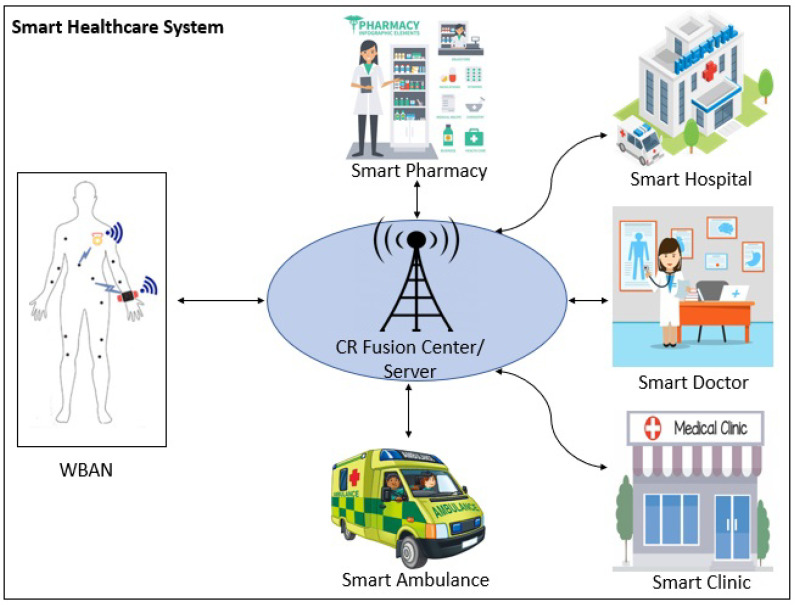
Cognitive radio-based smart healthcare system.

In the current technologically fast-paced world, people are facing many health-related issues and concerns. Therefore, it is the need of time for quick and reliable healthcare and monitoring systems. Smart healthcare is one of the remedies to address health-related issues and patient care remotely in real-time scenarios. Smart healthcare has gained the attention of researchers and industries dramatically in recent years [1,2,3,4]. Smart healthcare can make it very easy and convenient for medical personnel to share their information and suggestions on real-time data of the patient’s medical conditions and history in a short time. The electroencephalogram (EEG)-driven secure and reliable cognitive authentication system provides a solution to fix the security and privacy problems for an IoT-based healthcare system [5]. The effectiveness of existing diabetic foot ulcer (DFU) techniques can be enhanced by using a sensor-based remote patient monitoring (RPM) healthcare system [6]. The early detection of human health issues is very important for the better provision of cures. Different sensors in the IoT-based health system collects health-related data for the early detection and real-time monitoring of human health [7]. Cognitive radio (CR)-based smart healthcare is one of the most popular and important research areas nowadays. Hybrid optical camera communication (OCC) and Bluetooth low energy (BLE) are used to make an efficient smart healthcare system [8]. The system ensures that patients’ real-time electrocardiogram (ECG) data are transmitted to a remote monitoring system in an efficient way. The smart healthcare system can also be beneficial for providing maximum advantages of smart medicine to patients at their door step by exploiting the CR technology. Smart medicine uses different artificial intelligence (AI) techniques to process the patient’s health data on a micro level, even at the patient’s genetic level, and prescribes relevant treatments [9,10,11]. Cognitive sensors in CR-based smart healthcare continuously sense the available spectrum to transmit and share the patients’ data with the remotely placed server via CR base station(s) or a fusion center. An architecture of a CR-based smart healthcare system can be visualized in Figure 1. Different monitoring wireless sensors are attached to the human body. These sensors monitor the human body parts for which they are placed and collect the real-time data of the respective parts. These monitoring sensors are capable of performing spectrum sensing. Once they find a free spectrum, they share their collected data with a remotely placed fusion center or data server. Other components of the smart healthcare system, such as hospitals, ambulance services, pharmacies, and doctors’ clinics, are also equipped with CR technology and are connected to the server. When the monitoring sensors on the human body share their data with the server, these components also receive those data simultaneously through the server. Then, according to the level of health condition based on the sensors’ data, the respective component of the CR-based smart healthcare system responds to the patient and provides immediate and real-time advice and precautions.

There has been significant growth in the consumption of wireless spectrum bands in the past couple of decades. Cognitive radio (CR) has been under rigorous research to overcome spectrum scarcity and underutilization [12]. Application areas of cognitive radio can be smart healthcare systems, disaster relief, military and defense, emergency scenarios, industry, transportation and communication, internet of things (IoT), wireless body area networks (WBANs), and many more [13,14,15]. The throughput of the cognitive radio-enabled unmanned aerial vehicle (CR-UAV) is enhanced by jointly designing the UAV trajectory and resource allocation [16]. High data rates and minimal end-to-end routing delays in CR-based IoT communication are achieved by using a reinforcement learning (RL)-based routing approach [17]. The energy harvested communication protocol is a good approach to optimize the throughput of the UAV-assisted CR system [18]. A Q-learning-based dynamic spectrum access is considered in three different access scenarios, such as orthogonal multiple access (OMA), underlay spectrum access, and non-orthogonal multiple access (NOMA), to utilize the spectrum resources intelligently in the cognitive industrial internet of things (CIIoT) [19]. The fair and cooperative medium access control (FC-MAC) protocol enhanced the performance efficiency of the heterogeneous CR-based vehicular ad hoc network (VANET) [20,21].

The most important part of cognitive radio technology is spectrum sensing. The role of this part is to sense the spectrum and detect unused or free channels with the help of secondary users (SUs). From its beginning, researchers have been researching the development of different methods for spectrum sensing. Various methods have been proposed by the researchers. Energy detection, cyclostationary feature detection, and matched filter [22] are the most commonly used methods of spectrum sensing. The probability of detection ($P_{d}$) and probability of false alarm ($P_{f}$) are among the important parameters used in spectrum sensing. A high value of $P_{d}$ and lower value of $P_{f}$ are always required to avoid interference by SUs to PUs. There exists a trade-off between the optimal sensing time and spectrum hole utilization in cognitive radio networks [23]. A solution of an optimization problem maximizes the spectrum utilization efficiency of secondary users by considering the different possible communication scenarios of SUs in CRN [24]. Sampling controlled block orthogonal matching pursuit (SC-BOMP), schemes of wideband compressive spectrum sensing (CSS) provided the high sensing accuracy of CRNs in real time [25]. A convolutional neural network (CNN) obtained high spectrum detection accuracy under different noise models in CRN [26]. One of the main purposes of smart healthcare systems is to provide health-related facilities to people remotely. In smart healthcare systems, most of the monitoring devices and nodes share their data with remotely placed servers or physicians through a wireless medium, but spectrum allocation for new wireless services and applications is a big challenge for authorities. Therefore, cognitive radio technology can be used to overcome this issue. Motivated by the fact of spectrum scarcity and underutilization, we propose machine learning-based spectrum sensing in CR-based smart healthcare systems for the efficient use of the primary or existing spectrum for data sharing by secondary users without interfering with the primary users. In this paper, tree-based algorithms (TBAs) of machine learning are used to evaluate the spectrum sensing. To the best of our knowledge, these algorithms are yet to consider evaluating spectrum sensing.

This work has the following contributions:

1. Data set creation on simulated and theoretical values of $P_{d}$ and $P_{f}$ alarm.
2. Tree-based algorithms (TBAs), including fine tree, coarse tree, ensemble boosted tree, medium tree, ensemble bagged tree, ensemble RUSBoosted tree, and optimizable tree classifiers, are used to classify given data in MATLAB.
3. The evaluation of these classifiers’ performance measures is presented based on the training and testing accuracies. This evaluation is very helpful to obtain better results of spectrum sensing.
4. Minimum classification error (MCE) of optimizable tree is also plotted and discussed for both simulated and theoretical data sets.

The rest of the paper is composed of the following sections: Section 2 consists of the discussions on different works related to the involvement of machine learning/artificial intelligence and cognitive radio technologies in smart healthcare applications. A system model, discussing spectrum sensing and data sets creation for training and testing the models, is discussed in Section 3. Section 4 discusses the simulation and theoretical results to evaluate the performance of all classifiers used in this work. The paper concludes with Section 5.

## 2. Related Work: Smart Healthcare Using Machine Learning and Cognitive Radio Technologies

**Figure 2:**
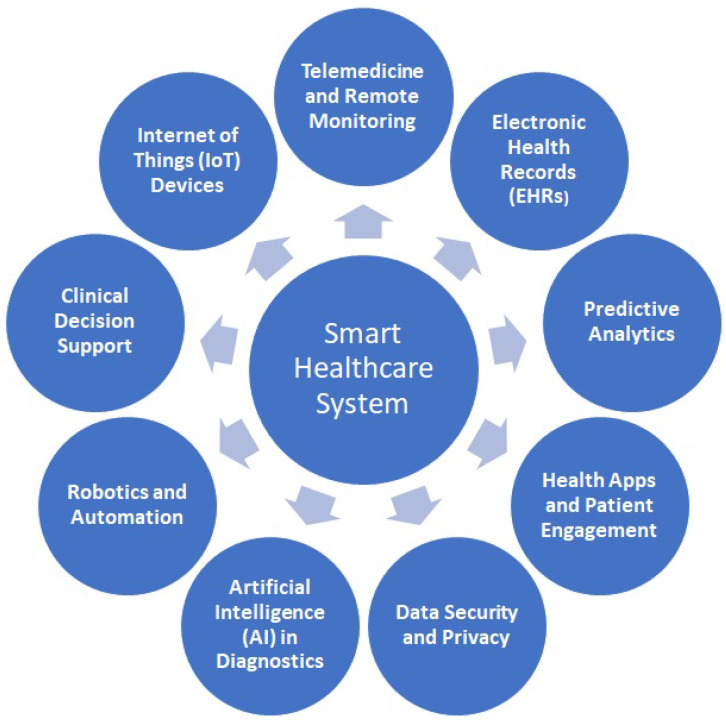
Advanced technologies as integral parts of smart healthcare system.

A smart healthcare system refers to the integration of advanced technologies, such as robotics and IoTs, data analytics, and intelligent algorithms of AI and ML, into the healthcare industry to improve patient care, streamline processes, and enhance overall efficiency of system. The efficient use of modern technologies can make smart healthcare systems superior and robust over the conventional healthcare systems. Smart healthcare systems can encompass a wide range of advanced technologies and concepts as shown in Figure 2.

Data storage and the management of patient health records collected by monitoring devices have significant importance in an efficient smart healthcare system. Healthcare professionals can access patient health record through EHRs. EHRs ensure better care coordination between the patients and their physicians to reduce chances of errors in the treatment [27]. Telemedicine and remote monitoring in smart healthcare system help patients to consult and seek medical care advice from their physicians remotely. Monitoring devices regularly monitor health metrics and send real-time data to physicians for proactive intervention [28,29]. Different wearable health monitoring devices in the IoT-based smart healthcare system collect data of patients’ health condition to provide personalized healthcare recommendations [30,31]. Smart healthcare systems can offer clinical decision support to healthcare professionals by providing evidence-based treatment recommendations and alerts about potential drug interactions or allergies. Robotic technologies can assist in surgeries, medication dispensing, and other medical tasks, enhancing precision and reducing the risk of human error [32]. Advanced data analytics and machine learning can predict disease outbreaks, patient needs, and trends. This helps healthcare providers to allocate resources effectively and make informed decisions. AI-based algorithms can be used to process medical images for faster and more accurate diagnosis of cancer or other diseases [33]. Mobile applications can encourage patient engagement by offering tools for medication reminders, exercise tracking, and lifestyle management. These applications can also provide access to health information and educational resources. As healthcare systems become more connected and reliant on data, robust security measures are essential to protect patient privacy and sensitive medical information. Smart healthcare systems are integrated with emergency services to provide real-time location data and medical information during emergencies, enabling faster and more effective responses. Analytics can help hospitals and healthcare facilities to optimize resource allocation, such as staff scheduling and bed availability, leading to improved patient flow and reduced wait times [34]. Comprehensive patient care also requires that different healthcare systems and devices can communicate and share data seamlessly with each other.

An efficient and successful smart healthcare system can be designed by incorporating machine learning and cognitive radio with it [35,36]. Smart healthcare devices and systems must be spectral and energy efficient while they are assisted by cognitive radio [37,38]. Spectrum utilization and energy harvesting protocols can make WBANs more convenient and efficient in smart healthcare systems and applications [39]. A patient-centric heterogeneous smart healthcare network predicts the patients’ health condition by employing different machine learning algorithms, including decision tree [40]. Data security and the timely evaluation of patients’ data in smart healthcare is the most important factor. A novel data encryption solution has made medical data transmission secure between CR-based devices and systems in a smart healthcare network [41]. 5G and 6G technologies have high bandwidth and data rates; therefore, these technologies can have a key role in the development of smart healthcare systems for the betterment of humanity. A comprehensive review on 5G and advanced technologies-based smart healthcare solutions is given in [42].

A very famous research area in computer science is machine learning that aims to create algorithms and software which can train and test the system for different data sets of interest and act intelligently while they are introduced to new information [43]. Machine learning techniques and algorithms are also used widely in the development of modern technologies, such as image processing, computer vision, speech recognition, and object (face, text, posture, and people) detection in robotics [44,45]. Considering the effective use of machine learning in other areas of science and technology, the healthcare system can also take advantages of ML to transform conventional healthcare systems into smart healthcare. The dream of smart healthcare systems can become true by adapting ML and CR technologies in such a way that sensing and monitoring devices in the healthcare system can adapt their parameters according to the dynamic radio conditions for real-time data transmission and processing. A deep learning (DL)-based convolutional neural network (CNN) model showed good performance for detecting the movement of a fractured ankle after surgery [46]. Machine learning (ML) has been used extensively over the years to predict and decide the spectrum availability in CRNs. In [47], the authors used support vector machines (SVMs) for joint spectrum sensing and spectrum allocation to network dynamics-aware IoT devices. The cost-effective and energy-efficient spectrum detection of real-time signals in a CRN is performed by using SVM, decision tree (DT) and KNN [48]. Different ML and DL techniques and algorithms, including decision trees, are used to predict human emotions that are positive, neutral, or negative from the EEG signals [49]. Decision tree classifier with other ML classifiers [50] are used for predicting the free spectrum. A decision tree-based energy efficient protocol has made it possible for decease detection in mobile healthcare network [51]. The efficiency of health-monitoring systems can be increased by the privacy-preserving decision tree (PPDT) classification scheme [52]. Three tree-based algorithms—random forest (RF), gradient boosting (GB), and extra trees (ET)—are used to examine the importance of several aspects of medical staff engagement in healthcare organizations [53]. A novel segment-based cognitive radio vehicle ad hoc network (CR-VANET) architecture can solve the spectrum scarcity problem by using fuzzy and naïve Bayes algorithms [54]. KMeans, AND and OR spectrum-sensing techniques are used to compare their performance in CRNs [55]. Q-learning-based spectrum sensing adaptively allocates the multimedia data over multiple spectrum holes [56]. A decentralized RL resource allocation scheme improves the spectrum utilization [57]. Ref. [58] provides the solutions to different spectrum sensing challenges by using different supervised and unsupervised machine learning algorithms. An intrusion of unauthorized data during cooperative spectrum sensing (CSS) can be avoided by K-medoids and mean-shift data fusion methods [59]. A comparison of several machine learning techniques, including K-nearest neighbors, naive Bayes, random forest, SVM, etc., for spectrum sensing is presented in [60].

## 3. System Model

**Figure 3:**
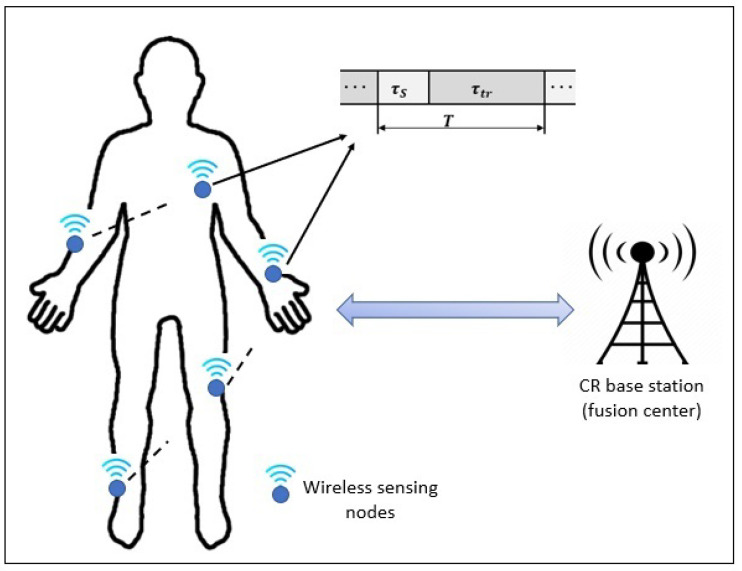
Working mechanism of cognitive radio in the smart healthcare system.

A working mechanism of cognitive radio in smart healthcare system is shown in Figure 3. Different sensing and monitoring nodes are implanted on the human body to monitor the health condition of different human body parts and perform spectrum sensing in the designated time slot to share the monitored data with other components of smart healthcare.

**Figure 4:**
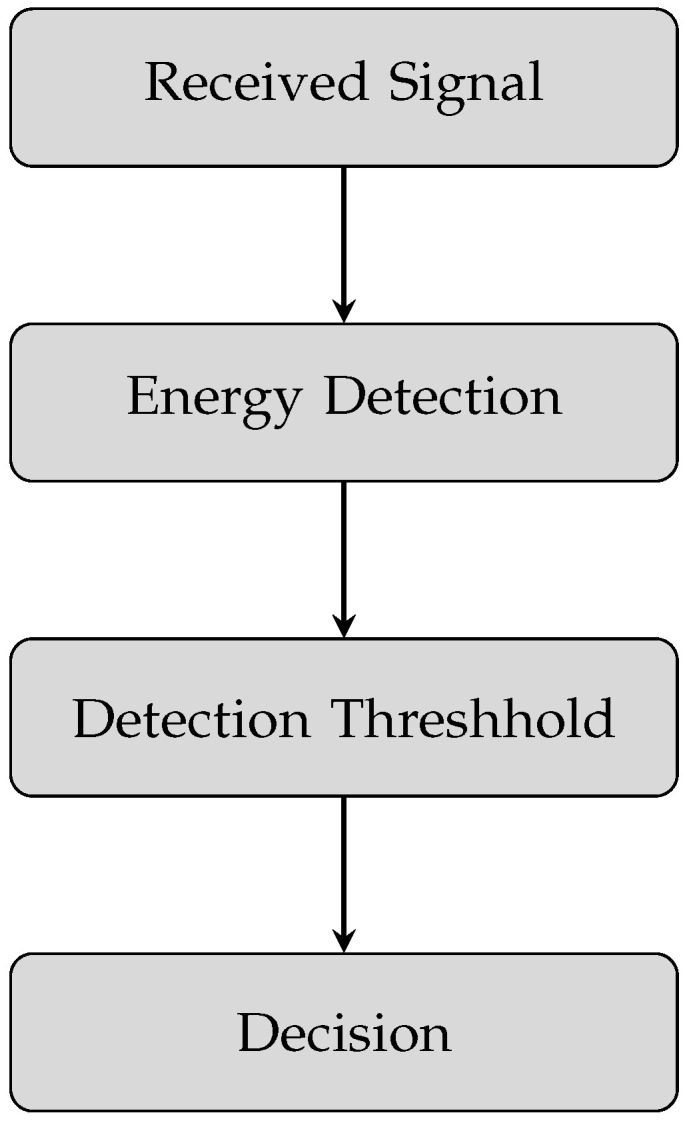
Spectrum sensing by using energy detection method.

Each sensing node has an equal time frame (*T*), which is further divided into two time slots, i.e., sensing ($\tau_{s}$) and transmission ($\tau_{tr}$) slots. In $\tau_{s}$, the slot-sensing nodes perform the spectrum sensing of the primary users and when it is declared that the spectrum is free to use, they transmit their data to the CR base station or fusion center in the $\tau_{tr}$ slot. On the other hand, the CR base station communicates with other components of the smart healthcare system to share the data received from the sensing nodes. The sensed data of secondary users are classified by using tree-based algorithms (TBAs), and the training and testing accuracy of the spectrum sensing is evaluated to decide whether the spectrum is free to use or not. Assume a centralized CR-based WBAN for a smart healthcare system which consists of M wireless sensing nodes, hereinafter secondary users (SUs). A binary hypothesis test can be applied to the received signal to perform the spectrum sensing by the SUs, which is expressed as

(1) $$y\left(n\right)=\left\{\begin{array}{cc} w(n) & H_{0}\\ s(n)+w(n) & H_{1}, \end{array}\right.$$

where s(n) represents the primary user’s signal with variance $\sigma_{s}^{2}$ and w(n) represents additive white Gaussian noise (AWGN) with zero mean and variance $\sigma_{n}^{2}$. $H_{0}$ and $H_{1}$ represent the binary hypothesis of the absence and presence of PU, respectively. There are several methods to perform spectrum sensing, but energy detection is the most used method due to its simplicity and no requirement of prior knowledge of the primary signal. In this paper, we also consider the energy detection method. The process of the energy detection-based spectrum sensing is shown in Figure 4.

Process shows that SUs collects N number of samples of received signal according to Equation (1) by continuously scanning the ambient environment. After collecting the required number of samples, the average energy of these received samples is calculated by taking the square of the magnitude of each sample and averaging the sum of all samples with the total number of received samples. A predefined detection threshold needs to be calculated, provided that a high target detection probability will be achieved. The average energy of the received samples is then compared with a predefined detection threshold. In the last step, the decision is made based on the comparison results of the average energy of the signal and detection threshold. If the average energy becomes greater than the detection threshold, it is decided that the primary user is present in the sensed spectrum; otherwise, the spectrum is declared free to use for SUs. The average energy of the received samples of the primary signal is known as test statistic T, which is used to compare with the detection threshold. T can be expressed as

(2) $$T=\frac{1}{N}\sum_{n=1}^{N}{\left|y\left(n\right)\right|}^{2}.$$

Probabilities of detection $\left(P_{d}\right)$ and false alarm $\left(P_{f}\right)$ are two important parameters associated with spectrum sensing and also show the performance of CRNs. For an efficient CRN, high $\left(P_{d}\right)$ and low $\left(P_{f}\right)$ are always required. $\left(P_{d}\right)$ actually gives us the probability of the presence of PU in the given spectrum and $\left(P_{f}\right)$ gives the wrong probability of the presence of PU in the given spectrum. Therefore, high $\left(P_{d}\right)$ is always required so that PUs cannot be interfered by SUs. On the other hand, $\left(P_{f}\right)$ is a missed opportunity to use the free spectrum, and therefore, low $\left(P_{f}\right)$ is needed for efficient CRNs. $\left(P_{d}\right)$ and $\left(P_{f}\right)$ are normally calculated by comparing T with the predefined detection threshold λ given that primary users are available or not in the spectrum. $P_{d}$ and $P_{f}$ in terms of T and λ can be defined as [61]

(3) $$P_{d}=Pr\left(T>\lambda |H_{1}\right),$$

(4) $$P_{f}=Pr\left(T>\lambda |H_{0}\right)$$

The test statistic T has chi-square under hypothesis $H_{0}$ and non-central chi-square distributions under $H_{1}$, with *N* degrees of freedom. It is assumed that each SU collects a large number of samples (N); therefore, according to the central limit theorem, the PDF of y(n) denoted as *f(y)* can be considered to be a Gaussian distribution and is given as [61]

(5) $$f\left(y\right)=\left\{\begin{array}{cc} N(\sigma_{n}^{2},\frac{\sigma_{n}^{4}}{N}) & H_{0}\\ N\left(\left(\gamma +1\right)\sigma_{n}^{2},\frac{\left(2\gamma +1\right)\sigma_{n}^{2}}{N}\right) & H_{1}, \end{array}\right.$$

where γ expresses the signal-to-noise ratio (SNR). The process of spectrum sensing starts by converting the received signal into N number of samples according to the requirement.

($P_{d}$) and ($P_{f}$) depend on N. A higher value of N guarantees higher ($P_{d}$) and lower ($P_{f}$). So, ($P_{d}$) and ($P_{f}$) can be expressed as follows [23]:

(6) $$P_{d}=Q\left(\left(\frac{\lambda }{\sigma_{n}^{2}}-\gamma -1\right)\sqrt{\frac{N}{2\gamma +1}}\right),$$

(7) $$P_{f}=Q\left(\left(\frac{\lambda }{\sigma_{n}^{2}}-1\right)\sqrt{N}\right),$$

where Q(·) is known as the Q-function, which is defined by

(8) $$Q\left(x\right)=\frac{1}{\sqrt{2\pi }}\int_{x}^{\infty }exp\left(-\frac{v^{2}}{2}\right)dv.$$

In spectrum sensing, a higher target detection probability ($\bar{P_{d}}$) is assumed such that there are fewer chances of missed detections of the presence of PU. The missed detection of the presence of PU causes interference with PU communication, which is undesirable in CRNs. Therefore, $\bar{P_{d}}$ can be used to obtain such a value of λ, which can be helpful to achieve higher ($P_{d}$) and lower ($P_{f}$):

(9) $$\lambda =\sigma_{n}^{2}\left(\gamma +1+\sqrt{\frac{2\gamma +1}{N}}Q^{-1}\left({\bar{P}}_{d}\right)\right).$$

Therefore, $P_{f}$ related to ${\bar{P}}_{d}$ can be computed as [23]

(10) $$P_{f}=Q\left(\sqrt{2\gamma +1}Q^{-1}\left({\bar{P}}_{d}\right)+\gamma \sqrt{N}\right).$$

**Figure 5:**
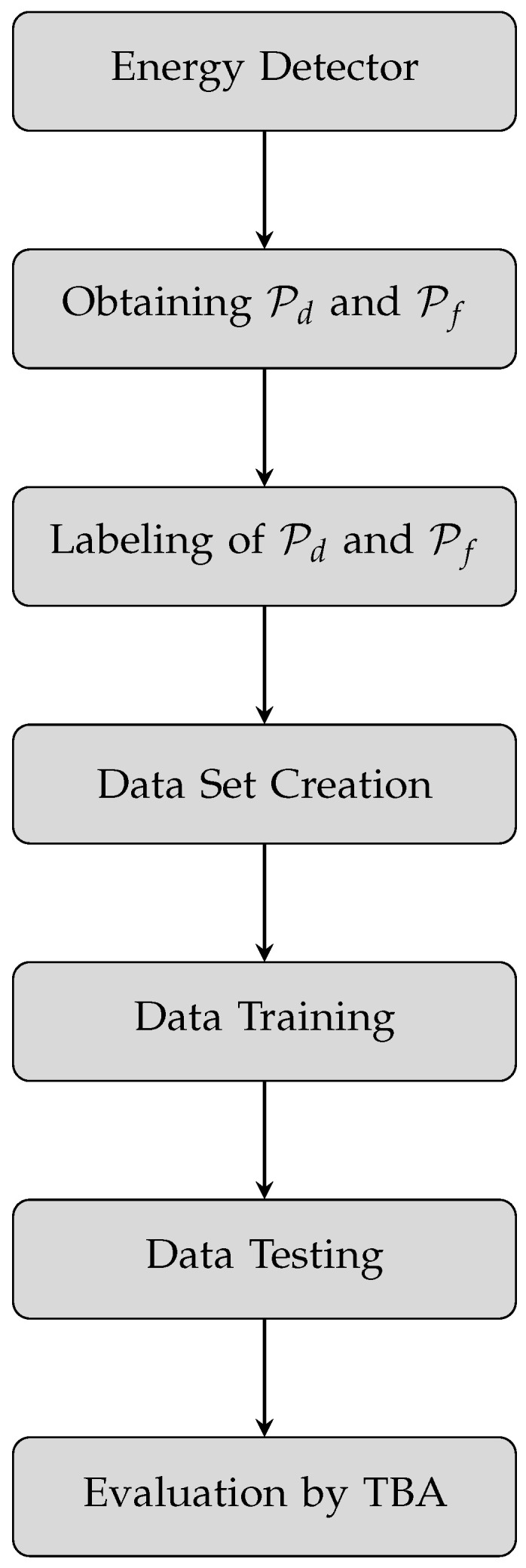
Proposed system flow diagram for both theoretical and simulated data sets.

Once the sensing results in the form of $P_{d}$ and $P_{f}$ are obtained, the above-mentioned tree-based algorithms are used to train and test the model to evaluate the spectrum sensing in the CR-based smart healthcare system. The flow diagram of the proposed system to evaluate the spectrum sensing for both theoretical and simulated data is shown in Figure 5.

In the given flow diagram, the energy detection block shows that a complete energy detection process as discussed earlier is applied to the received signal to compare the average energy of received signal samples with a pre-defined detection threshold. Based on the comparison results in the first block, the probabilities of detection and false alarm are calculated in the next block. The first two blocks show the conventional method of the spectrum-sensing process. Onward, these block applications of ML begin on the data. First, the values of $P_{d}$ and $P_{f}$ are labeled with ‘1’ and ‘0’, respectively. The labeled values of $P_{d}$ and $P_{f}$ are then fed to the data set creation block to create a data set of the two probabilities. In the data-training block, the model is trained for 70% of the values of the data set. After training the model, the remaining 30% of the data set is used to test the model. The results of the spectrum evaluation in the form of training and testing accuracies of tree-based algorithms (TBAs) are gathered after training and testing the model with those TBAs. A theoretical data set is created by using the numerical expressions of energy detection, $P_{d}$ and $P_{f}$, while the simulated data set is created by simulating the $P_{d}$ and $P_{f}$. Simulation is performed by comparing the average energy of randomly generated signal samples with a predefined detection threshold. Theoretical and simulated data sets now consist of theoretical and simulated labeled $P_{d}$ and $P_{f}$, respectively. Fine tree, medium tree, coarse tree [62], boosted tree [63], bagged tree [64], RUSBoosted tree [65,66], and optimizable tree [67] algorithms are used for classification. Tree base algorithms are easy to translate, fast to predict, and low in memory usage. Optimizable tree classification performs hyperparameter tuning by default using Bayesian optimization [68]. Training and testing accuracies of all algorithms are taken at the end to evaluate the spectrum sensing. Fine tree, medium tree, coarse tree and optimizable tree are types of decision tree (DT) classifiers of ML. DT is considered a nonparametric supervised ML algorithm which splits the input data (root node) into further sub data points known as branches, internal nodes and leaf nodes. The splitting of data in this way creates a tree-like shape which has roots, branches and leaves. DT learning uses the divide-and-conquer strategy to find the optimal splitting points in the input data by performing a greedy search. The working mechanisms of fine tree, medium tree, coarse tree, and optimizable tree are slightly different from each other. The main difference of these classifiers is the complexity of the tree they create by using different numbers of splits. The maximum numbers of splits for fine tree, medium tree, coarse tree, and optimizable tree are 100, 20, 4, and 2, respectively. All these classifiers employ the Gini diversity index to evaluate the accuracy of splits for random data points. Boosted tree, bagged tree, and RUSBoosted tree are types of ensemble ML methods. Ensemble learning combines the multiple decision tree algorithms, which show weak behavior in the classification of input data. All these classifiers use the decision tree learner with different numbers of splits. Boosted tree, bagged tree, and RUSBoosted tree have 20, 199, and 20 maximum number of splits, respectively.

## 4. Results and Discussion

In this section, the Matlab simulation results are provided to evaluate the performance of the proposed system. The necessary parameters to perform the spectrum sensing and to obtain the theoretical and simulated values of $P_{d}$ and $P_{f}$ are set as $f_{s}=6$ MHz, target detection probability ($\bar{P_{d}}$) = 0.9, and SNR (γ)=−15 dB. The analysis of the classification learning tool in Matlab [69] is used to perform spectrum-sensing data evaluation.In Matlab, we create an environment by generating the samples of random noise and primary signal. In total, 1000 Monte Carlo simulations are performed to generate noise and primary signal separately. In each simulation, the average energy of received signal is calculated for both noise only and noise + primary signal. The average energy of both scenarios is compared with the predefined energy detection threshold. After 1000 simulations, the number of times that the average energy is greater than the threshold under the hypothesis $H_{0}$ (only noise)) is divided by the total number of simulations, and the result is considered $P_{f}$. In this way, 200 values of $P_{f}$ are calculated. Similarly, $P_{d}$ is calculated by dividing the number of times that the average energy is greater than the threshold under the hypothesis $H_{1}$ (noise + primary signal) with the total number of simulations. In this way, the simulated values of $P_{d}$ and $P_{f}$ are calculated. Theoretical values of both probabilities are calculated by using their mathematical expressions, which are described in the relevant section.

### 4.1. Data Modeling

There are two different data sets created from the spectrum-sensing process. Both simulated and theoretical values of the probability of detection ($P_{d}$) and probability of false alarm $P_{f}$ are passed through the labeling process. Values of $P_{d}$ are labeled with ‘1’, while the values of ($P_{f}$) are labeled with ‘0’, respectively, to create the two data sets of both simulated and theoretical probabilities. The default value of cross validation is set as 5. In total, 70% of the data of both the simulated and theoretical data sets is used to train the classifiers, while the remaining 30% is used for testing purposes.

### 4.2. Results of Classifiers

After training and testing in the classification learner, all tree-based models are compared to evaluate their performance in terms of training (validation) and testing accuracy. A comparison of the training and testing accuracies of different tree-based classifiers for simulated data for 1000, 1500, 2000, and 2500 samples of the received signal is shown in Table 1.

The results in the table show that the training and testing accuracies of all classifiers increase with the increase in the number of received signal samples. This is because when the number of samples increases, the sensing results also change in the form of $\left(P_{d}\right)$ and $\left(P_{f}\right)$. The higher number of samples provides high $\left(P_{d}\right)$ and low $\left(P_{f}\right)$, which are actually the desired results expected from efficient spectrum sensing. So when the high $\left(P_{d}\right)$ and low $\left(P_{f}\right)$ are labeled with ‘1’ and ‘0’ respectively, it provides more 1s than 0s in the data set. Therefore, classifiers are trained with the data set which has a maximum number of the same labels, i.e., 1s, and as a result this provides high training and testing accuracies. Among all classifiers, optimizable tree provides better training and testing accuracies for each given number of the received signal’s samples. The training and testing accuracies of optimizable tree are 89.30% and 86.70% for 1000 samples, 92.90% and 91.70% for 1500 samples, 96.40% and 96.70% for 2000 samples, and 98.60% and 96.70% for 2500 samples, respectively.

Table 2 shows the comparison of training and testing accuracies of all classifiers for the same theoretical data set for 1000, 1500, 2000, and 2500 numbers of received signal samples.

The trend in the values of training and testing accuracies is the same as that discussed in Table 1 for simulated data, i.e., the higher the number of samples, the higher the training and testing accuracies. Optimizable tree outperforms all other classifiers in terms of training and testing accuracies for all numbers of samples. Optimizable tree gives 87.10%, 93.60%, 94.30%, and 98.00% training accuracies and 85.00%, 91.70, 95.00% and 95.00% testing accuracies for 1000, 1500, 2000, and 2500 samples, respectively. The reason in the increment of these accuracies with the increase in the number of samples is the same as that discussed in Table 1 for simulated data. There are significant similarities between the training and testing accuracies of both simulated and theoretical data.

A comparison of the testing accuracies of the proposed system with existing works is shown in Table 3.

The average testing accuracies of both the theoretical and simulated data sets of the proposed optimizable tree classifier are 95% and 96%. Tri-agent reinforcement learning [54] and unsupervised deep spectrum sensing [70] classifiers provide 94% and 86% theoretical testing accuracies of spectrum sensing, respectively. Similarly, some other classifiers like the back-propagation neural network [58], ensemble machine learning [71], and minimum covariance determinant [72], provide 90%, 89%, and 89.8% simulated testing accuracies, respectively. The results show that optimizable tree outperforms the other classifiers with respect to both the theoretical and simulated testing accuracies.

### 4.3. Minimum Classification Error (MCE)

The goal of the MCE plot is to reduce the resulting classification error when attempting to classify a new information set. Usually, these classifications use some structure of the statistical model to describe the information. The x label shows the number of iterations, and the y label shows the minimum classification error. The minimum classification error has the following details [69]:

- Estimated minimum classification error: Each blue element corresponds to the subdivision error estimate combined with the optimization process when taking into account all the parameter value units. Estimation is primarily based on the high self-assurance of the current goal model of the divisions.
- Minimum error of classification: Each circle corresponds to a fixed-phase calculation error that is combined over a long distance using a fine-tuning process.
- Hyperparameter point: The rectangle shows the generation corresponding to the best point hyperparameters.
- Error of hyperparameters. The feature indicates an error in the classification phase.

**Figure 6:**
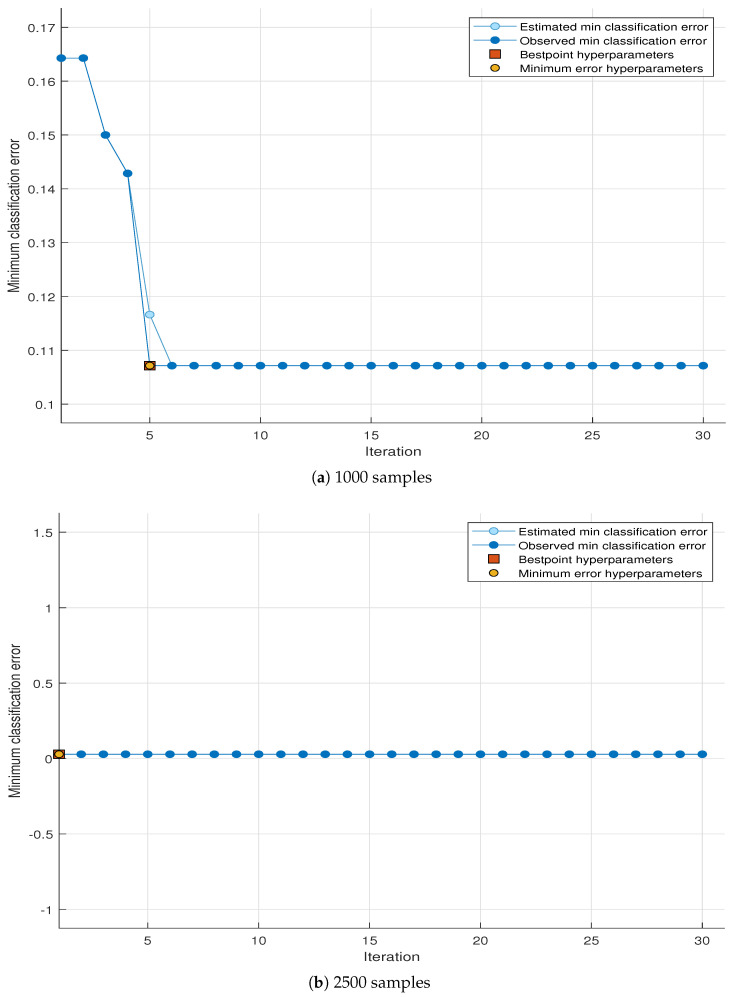
MCE plot of simulated data at different number of samples.

**Figure 7:**
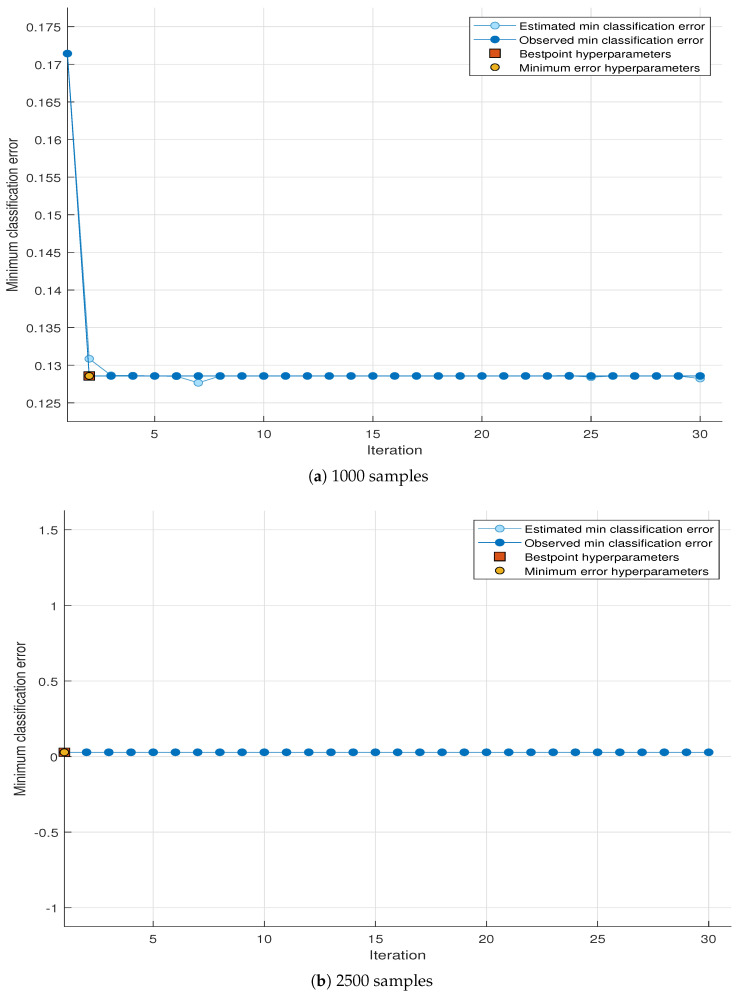
MCE plot of theoretical data at different numbers of samples.

Figure 6 and Figure 7 show the minimum classification error (MCE) plot of the optimizable tree of both the simulated and theoretical data sets for 1000 and 2500 numbers of received signal samples, respectively. Four different MCE values, i.e., estimated minimum classification error, observed minimum classification error, best point hyperparameters, and minimum error hyperparameters, are shown in each MCE plot. Figure 6a shows the graph of the MCE plot of simulated data for 1000 samples, with the minimum classification error shown on the y-axis and number of iterations on the x-axis. The estimated MCE value is 0.164 at the start and becomes constant at 0.108 after the 6th iteration. The observed MCE also starts from 0.164 and becomes constant at 0.108 after the 6th iteration. Both estimated and observed MCEs are almost completely overlapped with each other, except for the estimated MCE value of 0.117 at the 5th iteration, which shows the success of the classification process of optimizable tree. The best point hyperparameters and minimum error hyperparameters are 0.108 at the 5th iteration, which shows that the optimization of the hyperparameter point in the hyperparameter search range exhibits less error to enhance the performance of the learning process for optimizable tree. Similarly, in Figure 6b, the graph of the MCE plot of the simulated data set is shown for 2500 samples. The observed and the estimated MCEs are constant for all iterations and are very close to zero. The best point hyperparameters and minimum error hyperparameters also show a smaller value close to zero in the very beginning of the iterations.

Figure 7a,b show the results of MCE for the theatrical data set for 1000 and 2500 samples, respectively. The MCE plot for 1000 samples shows that the initial values of the estimated and observed MCEs start to form 0.172 at the first iteration and become constant after the 2nd iteration with value of 0.129. The common value of both best point hyperparameters and minimum error hyperparameters is 0.129 at the 2nd iteration. All MCE parameters for the simulated data set are also close to zero for 2500 samples like the MCE plot of the theoretical data set for 2500 samples.

All sub-figures in both Figure 6 and Figure 7 show that the estimated and observed minimum classification errors have higher values at the start of the iterations, but their values go down near the horizontal axis with the increase in iterations. The best hyperparameters point and minimum error hyperparameters remain at the lowest constant near the horizontal axis in all sub-figures for both simulated and theoretical data sets. It is clear from the figures that all MCE values remain at the lowest constant level with the increase in the number of samples. This is because when the number of samples increases, $P_{d}$ touches the highest value of 1 and $P_{f}$ goes down almost equal to 0. This results in better spectrum sensing, and the classifier obtains the true value of the presence of PU, and as a result, it gives higher accuracy values and fewer classification errors. It is also observed from the MCE plots of both simulated and theoretical data sets that the number of samples has a prominent influence on MCE. When the number of samples increases, the MCE parameters exhibit lower values, which shows that the optimizable tree performance increases from the lower to higher number of samples. Both simulated and theoretical MCE plots show close similarity in the values.

## 5. Conclusions

Smart healthcare is one of the hot research topics by researchers nowadays, for which different on-body and off-body sensors and devices monitor and share patients’ data with healthcare personnel and hospitals for quick and on-time decisions about patient health. The smart healthcare system has the potential to revolutionize healthcare delivery, making it more personalized, efficient, and accessible while improving patient outcomes and the overall quality of care. However, it also requires careful implementation, the consideration of ethical concerns, and continuous monitoring to ensure its effectiveness and security. Cognitive radio (CR) is meant to solve the problem of spectrum scarcity for different wireless services and applications. CR technology can be useful for smart healthcare systems to send and receive the monitoring data through the PU spectrum without interfering with the PU communication. CR-based smart healthcare resolves two main issues: First, it allows monitoring sensors (secondary users) to transmit data wirelessly when the primary user is inactive in the spectrum according to the spectrum sensing results. Second, it improves the use of spectrum utilization efficiency. In this paper, tree-based algorithms (TBAs) are used to evaluate the spectrum sensing efficiently. The energy detection method is used to decide the availability of the spectrum of interest. Data sets are created based on simulated and theoretical values of $P_{d}$ and $P_{f}$. Different classifiers of machine learning like fine tree, coarse tree, ensemble boosted tree, medium tree, ensemble bagged tree, ensemble RUSBoosted tree, and optimizable tree are used to calculate the training and testing accuracy-based sensing results. Comparisons of all said classifiers are made for both the simulated and theoretical results of $P_{d}$ and $P_{f}$. The investigation of all classifiers for the various number of samples is performed in terms of the training and testing accuracies and the minimum classification error (MCE) to compare the effectiveness of the proposed work for the better evaluation of spectrum sensing. Among all classifiers, optimizable tree provides better training and testing accuracies for each given number of the received signal’s samples. The training and testing accuracies of optimizable tree are 89.30% and 86.70% for 1000 samples, 92.90% and 91.70% for 1500 samples, 96.40% and 96.70% for 2000 samples, and 98.60% and 96.70% for 2500 samples, respectively. A comparison of our work with other ML and DL techniques will be presented in the extended version of this paper in our future work.

## Figures and Tables

**Table 1:** Comparison of tree-based classifiers for simulated data.

Classifiers	1000 Samples		1500 Samples		2000 Samples		2500 Samples	
	**Training Accuracy (%)**	**Testing Accuracy (%)**	**Training Accuracy (%)**	**Testing Accuracy (%)**	**Training Accuracy (%)**	**Testing Accuracy (%)**	**Training Accuracy (%)**	**Testing Accuracy (%)**
Fine Tree	85.00	85.00	92.10	91.70	94.30	93.30	97.10	96.70
Medium tree	85.00	85.00	92.10	91.70	94.30	93.30	97.10	96.70
Coarse Tree	88.60	85.00	92.10	91.70	95.70	93.30	97.10	96.00
Boosted Trees	76.40	80.00	88.60	90.00	83.60	93.30	90.00	95.00
Bagged Trees	83.60	80.00	89.30	90.00	93.60	93.30	97.10	95.00
RUSBoosted Trees	75.70	76.70	88.60	88.30	83.60	93.30	90.00	95.00
Optimizable Tree	89.30	86.70	92.90	91.70	96.40	96.70	98.60	96.70

**Table 2:** Comparison of tree-based classifiers on theoretical data.

Classifiers	1000 Samples		1500 Samples		2000 Samples		2500 Samples	
	**Training Accuracy (%)**	**Testing Accuracy (%)**	**Training Accuracy (%)**	**Testing Accuracy (%)**	**Training Accuracy (%)**	**Testing Accuracy (%)**	**Training Accuracy (%)**	**Testing Accuracy (%)**
Fine Tree	82.90	85.00	90.00	88.30	93.60	93.30	97.10	95.00
Medium tree	82.90	85.00	90.60	88.30	93.60	93.30	97.10	95.00
Coarse Tree	83.60	85.00	91.40	90.00	93.60	95.00	97.10	95.00
Boosted Trees	71.40	75.00	75.70	85.00	82.10	90.00	87.10	93.30
Bagged Trees	77.90	75.30	89.30	85.00	92.90	90.00	95.70	93.30
RUSBoosted Trees	73.60	80.00	80.40	85.00	84.30	85.00	87.10	91.70
Optimizable Tree	87.10	85.00	93.60	91.70	94.30	95.00	98.00	95.00

**Table 3:** Comparison between proposed and existing systems with different classifiers.

Classifiers	Accuracy (Theoretical)	Accuracy (Simulated)
Optimizable Tree [proposed]	95%	96%
Tri-Agent Reinforcement Learning (TARL) [54]	94%	–
Unsupervised Deep Spectrum Sensing (UDSS) [70]	86%	–
Back-Propagation Neural Network (BPNN) [58]	–	90%
Ensemble Machine Learning (EML) [71]	–	89%
Minimum Covariance Determinant (MCD) [72]	–	89.8%

## Data Availability

Not applicable.

## Abbreviations

The following abbreviations are used in this manuscript:

CR	Cognitive radio
CRN	Cognitive radio network
SU	Secondary user
PU	Primary user
SS	Spectrum sensing
CSS	Cooperative spectrum sensing
TBA	Tree-based algorithm
MCE	Minimum classification error
OCC	Optical camera communication
BLE	Bluetooth low energy
ECG	Electrocardiogram
IoT	Internet of things
WBAN	Wireless body area network
UAV	Unmanned aerial vehicle
RL	Reinforcement learning
CI-IoT	Cognitive industrial internet of things
OMA	Orthogonal multiple access
NOMA	Non-orthogonal multiple access
FC-MAC	Fair and cooperative medium access control
SC-BOMP	Sampling-controlled block orthogonal matching pursuit
CSS	Compressive spectrum sensing
CNN	Convolutional neural network
DL	Deep learning
ML	Machine learning
SVM	Support vector machine
VANET	Vehicle ad hoc network
KBL	Kernel-based learning
ROC	Receiver operating characteristic
KNN	K-nearest neighbors
AWGN	Additive white Gaussian noise
PDF	Probability density function
SNR	Signal-to-noise ratio
AI	Artificial intelligence
